# Pressure Anesthesia

**Published:** 1917-04

**Authors:** 


### PRESSURE ANESTHESIA

The field must first be isolated. If the tooth is broken down so that a rubber dam can not be placed on it a copper band should be cemented to place. This will also facilitate in keeping the solution from leaking under the pressure. After cleaning and sterilizing the cavity, place a pellet of cotton saturated with the solution in the cavity. Then form a plug of base plate gutta percha the approximate form of the cavity and stick it to a large sized amalgam plugger. Then moisten the end of the gutta percha with the oil of cajuput and warm the end, but not the whole form. This softens the end of the gutta percha so that it seals the periphery of the cavity and the solution will not leak. A slow steady pressure held for about a minute will insure results. Of course, if there is no exposure it may take a second application after exposing the pulp.

The indications for the use of pressure anesthesia are all cases where extirpation is necessary and there is no infection. If infection is present conductive anesthesia must be used.—L. E. Bryant, in *Review*.

